# Role of nuclear receptor corepressor RIP140 in metabolic syndrome^☆^

**DOI:** 10.1016/j.bbadis.2010.12.016

**Published:** 2011-08

**Authors:** Meritxell Rosell, Marius C. Jones, Malcolm G. Parker

**Affiliations:** Institute of Reproductive and Developmental Biology, Imperial College London, Faculty of Medicine, Hammersmith Campus 158 Du Cane Road, W12 0NN, UK

**Keywords:** AR, androgen receptor, ATF, activating transcription factors, CBP, CRE-binding protein, CIDEA, cell death-inducing DNA fragmentation factor alpha-like effector A, CPT, 1β carnitine palmitoyltransferase 1β, CREB, CRE-binding protein, CtBP, C-terminal binding protein, Dnmt, DNA methyltransferase, EDL, extensor digitorum longus, ERR, estrogen related receptor, FA, fatty acid, FAS, fatty acid synthase, FOXC2, forkhead-box C2, GLUT4, glucose transporter 4, GR, glucocorticoid receptor, HDAC, histone deacetylase, HFD, high-fat diet, IL, interleukin, JNK, Janus N-terminal kinase, MEF, myocyte-specific enhancer binding factor, NCoR, nuclear receptor corepressor, NF-κB, nuclear factor κB, NR, nuclear receptor, NRF, nuclear respiratory factor, PCOS, polycystic ovary syndrome, PEPCK, phosphoenolpyruvate carboxykinase, PGC, PPARγ coactivator, PPAR, peroxixome proliferator-activated receptor, PRDM16, positive regulatory domain-containing 16, RAR, retinoic acid receptor, RBP4, retinol binding protein 4, ROS, reactive oxygen species, RXR, retinoid X receptor, SMRT, silencing mediator of retinoic acid and thyroid hormone receptor, SRC, steroid receptor coactivator, SREBP1c, sterol regulatory element binding protein 1c, TAG, triacylglyceride, TLR, Toll-like receptor, TNFα, tumour necrosis factor α, TR, thyroid hormone receptor, UCP1, uncoupling protein 1, VLDL, very low density lipoprotein, Metabolic syndrome, RIP140, White adipose tissue, Inflammation

## Abstract

Obesity and its associated complications, which can lead to the development of metabolic syndrome, are a worldwide major public health concern especially in developed countries where they have a very high prevalence. RIP140 is a nuclear coregulator with a pivotal role in controlling lipid and glucose metabolism. Genetically manipulated mice devoid of RIP140 are lean with increased oxygen consumption and are resistant to high-fat diet-induced obesity and hepatic steatosis with improved insulin sensitivity. Moreover, white adipocytes with targeted disruption of RIP140 express genes characteristic of brown fat including CIDEA and UCP1 while skeletal muscles show a shift in fibre type composition enriched in more oxidative fibres. Thus, RIP140 is a potential therapeutic target in metabolic disorders. In this article we will review the role of RIP140 in tissues relevant to the appearance and progression of the metabolic syndrome and discuss how the manipulation of RIP140 levels or activity might represent a therapeutic approach to combat obesity and associated metabolic disorders. This article is part of a Special Issue entitled: Translating nuclear receptors from health to disease.

## Research Highlights

► RIP140 regulates energy expenditure and storage in adipose tissue. ► RIP140 modulates muscle fiber type. ► RIP140 affects inflammatory responses. ► RIP140 is a promising target for treatment of metabolic disorders.

## 1 Introduction

Obesity has reached worldwide epidemic proportions, especially in Western countries, often associated with metabolic dysfunctions such as insulin resistance, dyslipidaemia, cardiovascular disease and even some cancers [1–3]. The clustering of these metabolic dysfunctions is recognized as the metabolic syndrome and the component disorders are essentially caused by a positive energy imbalance, where nutrient uptake is greater than energy expenditure. Strong epidemiological links between obesity, type II diabetes and cardiovascular diseases have suggested a common molecular pathogenesis. A limit on adipose tissue expandability has been proposed to provide a unifying causal link to the development of metabolic syndrome [4–6]. Challenging the body with nutrient excess for a prolonged period of time results in lipid deposition in adipose tissue. Adipose tissue expands in order to accommodate the excess of energy with adipocytes undergoing hypertrophy (increase in size) and hyperplasia (increase in number) to fulfil the increased demand for lipid storage. Hypertrophic adipocytes have an altered profile of adipokine secretion: a decrease in hormones/cytokines that promote insulin sensitivity, including adiponectin, and an increase in those that promote insulin resistance, such as resisitin and retinol binding protein (RBP) 4, and inflammation, such as interleukin (IL) 6 and tumour necrosis factor (TNF) α [7–9]. These inflammatory molecules recruit and activate macrophages present in the tissue to trigger low-grade inflammation in adipose tissue. Furthermore, activated macrophages block preadipocyte recruitment and limit hyperplasic expansion of the adipose tissue. Once the storage capacity of the adipose tissue is exceeded, lipids start to accumulate in non-adipose tissues, leading to lipotoxicity [10,11]. Steatosis of the liver, muscle, pancreas, heart, and kidney can lead to organ failure or exacerbate whole-body insulin resistance. In addition, increased levels of circulating free fatty acids (FAs) and development of type II diabetes are major risk factors for development of atherosclerosis and cardiovascular complications [12].

Several nuclear receptors (NRs), including peroxisome proliferator-activated receptors (PPARs), thyroid hormone receptors (TRs) and estrogen-related receptors (ERRs), have emerged as important metabolic regulators [13]. These receptors control the transcription of genes involved in metabolic pathways underpinning the pathophysiology of the metabolic syndrome. The ability of NRs to regulate gene transcription depends on the recruitment of coactivators and corepressors that remodel chromatin in the vicinity of the promoters. Both the expression and activity of cofactors are subject to regulation by a number of signalling pathways in a tissue-specific manner. The activity of many receptors is suppressed in the absence of ligand passively, in complexes with heat shock proteins in the cytoplasm, as in the case of androgen receptor (AR) and glucocorticoid receptor (GR) [14,15]. Some NRs, including TR, retinoid X receptor (RXR) and PPARs, can be actively repressed by recruitment of corepressors, such as nuclear receptor corepressor (NCoR) and silencing mediator of retinoic acid and thyroid hormone receptor (SMRT) [16,17]. Ligand binding results in conformational changes which lead to the dissociation of repressive complexes and the recruitment of coactivators, such as p300/CBP, p160/steroid receptor coactivator (SRC) and PPARγ coactivator (PGC) families, to activate transcription [18,19]. Ligand binding may not only result in the activation of subsets of genes but also the repression of certain genes. Receptor interacting protein (RIP) 140 was found to function as a corepressor for a number of ligand bound nuclear receptors with a role in metabolism (TR2, PPARs, LXRα, GR, retinoic acid receptor (RAR)/RXR) [20–25]. RIP140 is itself highly expressed in metabolic tissue like adipose tissue, skeletal muscle and liver [26]. Genome-wide expression arrays studies have highlighted its role as a global regulator of energy balance, repressing multiple metabolic pathways to reduce energy expenditure. On the other hand, recent studies have shown that RIP140 also functions as a coactivator for NR [22,27] or other types of transcription factors [28,29]. The molecular basis for these opposing functions is poorly understood and this review will focus on the biological roles of RIP140 in metabolism and inflammation and considers how targeting its function may provide an approach to treatment of metabolic syndrome.

## 2 Role of RIP140 in adipose tissue

Both white (WAT) and brown (BAT) adipose tissue share many common features such as their ability to take up glucose, synthesize and store triacylglycerides (TAG), and mobilize those TAG in situations of energy demand. However, they play largely opposing roles in mammalian physiology. WAT serves as the prime reservoir for storing excess caloric energy in the form of TAG. It is also an important source of endocrine hormones involved in metabolic regulation such as leptin, adiponectin, and resistin [7,30]. In contrast, BAT is the major site of adaptive thermogenesis capable of generating heat from fatty acid oxidation to maintain body temperature, particularly in response to cold exposure [31]. Thus, it is a net consumer of caloric energy. BAT is also a site of diet-induced thermogenesis [31]. The function of brown adipocytes is critically dependent on the expression and activity of uncoupling protein (UCP) 1, a mitochondrial protein almost exclusively found in BAT and therefore considered as a key molecular marker [31]. That WAT and BAT play an important role in the maintenance of energy homeostasis is reflected in the observation that small alterations in their function can have a large impact on whole-body metabolism [31]. Moreover, the ability of white and brown adipocytes in each depot to reversibly switch into one another has been reported [30,32,33], but the extent to which this occurs and the precise mechanisms involved are not fully understood (reviewed in [34]). The search for regulators that could mediate conversion of white adipocytes (energy storing) into brown adipocytes (energy consuming) has led to the identification of PGC1α, forkhead-box (FOX) C2 and positive regulatory domain-containing (PRDM) 16 as transcriptional regulators that have been found to promote a brown fat genetic program, while retinoblastoma protein and RIP140 have been described to favour a white adipose phenotype (reviewed in refs. [35,36]).

**Fig. 1:**
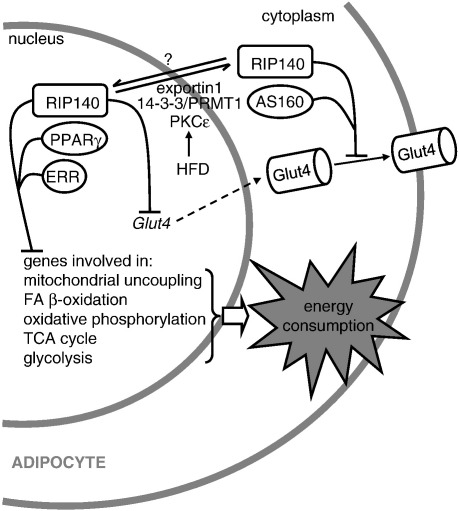
Actions of RIP140 in adipocytes. Nuclear RIP140 is recruited by nuclear receptors to repress sets of genes that promote energy consumption. *Glut4* gene is also transrepressed and the action of Glut4 protein is inhibited by cytoplasmic RIP140, contributing to insulin resistance. Cytoplasmic translocation of RIP140 is stimulated by PKCε-mediated phosphorylation, followed by 14-3-3-dependent recruitment of PRMT1, arginine methylation and export through exportin1. This sequence of post-translational modifications is promoted under a high-fat diet. PKCε, protein kinase Cε; PRTM1, protein arginine methyl transferase 1; HFD, high-fat diet.

RIP140 is most highly expressed in WAT where it regulates the expression of many genes involved in catabolic pathways (energy consuming), especially those involved in lipid and glucose metabolism. Mice devoid of the coregulator express higher levels of genes that regulate fatty acid oxidation and proteins involved in energy dissipation, highlighting its role as a corepressor and its role controlling the balance between energy consumption and energy expenditure [26]. On the contrary, genes involved in FA synthesis and gluconeogenesis seem to be downregulated in the absence of RIP140. As a consequence, RIP140 null mice are lean, with a 70% reduction of body fat and a 20% reduction in body weight compared to wild-type mice and they are resistant to high-fat diet (HFD) induced obesity and hepatic steatosis so that the mice maintain their insulin sensitivity [26,37]. It is particularly noteworthy that in the RIP140 KO animals WAT expresses genes characteristic of BAT including UCP1, fatty acid transporter carnitine palmitoyltransferase (CPT-1β) and the lipid droplet protein cell death-inducing DNA fragmentation factor alpha-like effector A (CIDEA). Targeted disruption in mice of several genes directly involved in energy metabolism and fat accumulation (LXRα, ERRα and retinoblastoma protein) also leads to a lean phenotype with a marked increase in UCP1 expression in adipocytes, particularly in white fat depots (see ref. [38] for a review). RIP140 expression is very low in preadipocytes and induced upon differentiation [37,39,40]. Studies in different adipocyte cell models (3T3-L1, MEFs (mouse embryonic fibroblasts), immortalised white adipocytes) have shown that RIP140 is not required for adipocyte differentiation. Analysis of gene expression profiles from the RIP140 null cell lines show that, RIP140 modulates the expression of similar genes involved in fatty acid and carbohydrate metabolism found in WAT, indicating that it functions as a cell autonomous corepressor. Most of the upregulated genes in the RIP140-null adipocytes are genes involved in catabolic pathways including fatty acid oxidation, oxidative phosphorylation, glycolysis and the tricarboxilic acid cycle (Fig. 1). Again, some genes normally restricted to expression in BAT, including UCP1, CIDEA and CPT1-β, are elevated in RIP140-null adipocytes [37,39]. Many of the genes regulated by RIP140 are also targets for the coactivator PGC1α that was originally identified in BAT as factor that is elevated in response to cold. PGC1α is a key regulator of mitochondrial biogenesis and oxidative metabolism in tissues with high oxidative capacity such as skeletal muscle, liver and brown fat by acting as a coactivator for a number of different transcription factors involved in metabolic control [41]. Since RIP140 also can bind to and repress many of these transcription factors, including PPARα, PPARγ and ERRα [21,39] it appears that the PGC1α and RIP140 coregulators may play mutually opposing roles in controlling sets of metabolic genes. We have found that both coregulators can be recruited simultaneously to the CIDEA promoter and it's noteworthy that they are able to physically interact. These observations suggest that RIP140 may be acting as a transcriptional brake that could be overcomed under conditions when the expression or activity of the coactivator is increased [42].

Recent studies by Li Na Wei and her colleagues indicate that RIP140 may not only be a transcriptional coregulator but may also function in the cell cytoplasm. They have found that cytoplasmic RIP140 inhibits glucose metabolism by reducing insulin-stimulated glucose transporter 4 (GLUT4) trafficking and glucose uptake [43] (Fig. 1). Importantly, the same study shows that high-fat feeding results in cytoplasmic localization of RIP140 in epididymal white adipocytes, highlighting the biological relevance of a function for RIP140 in the cytoplasm. The cytoplasmic role of RIP140 is in addition to the direct regulation of GLUT4 mRNA expression by RIP140 in mouse and human adipocytes [37,40]). These findings provide the basis for a novel mechanism by which RIP140 might impair glucose utilization and promote insulin resistance. The observations also suggest that irrespective of RIP140 expression levels it may also be important to establish whether there are changes in compartmentalization of the protein.

Very few studies have been carried out in humans. A decrease in RIP140 mRNA in biopsies of visceral WAT depots from obese patients has been reported with a strong correlation between body mass index and RIP140 mRNA levels [44]. It is conceivable that decreased levels of RIP140 serve as a compensatory mechanism to favour energy expenditure to reduce fat accumulation. In another study no difference was found in RIP140 expression between obese and lean women with polycystic ovary syndrome (PCOS), or between obese PCOS and lean controls [45]. Finally, a recent study shows that RIP140 is decreased in subcutaneous adipose tissue of obese women and increased by weight loss. In the same study, in primary culture of human adipocytes, RIP140 expression increased during adipocyte differentiation and its knockdown increased basal glucose transport and mRNA levels of GLUT4 and UCP1, a similar behaviour to that of the mouse ortholog [46]. Overall, high levels of human RIP140 in WAT of lean subjects may minimise energy utilization from depleted fat stores. At first sight, the overexpression of RIP140 in tissues from obese individuals would be predicted from the mouse studies, where its absence promotes reduced TAG accumulation; but on the other hand, the subcellular localisation of RIP140 was not examined in these studies and we have yet to elucidate signalling pathways that may control of the activity of RIP140 by post-translational modifications.

RIP140 is highly expressed in BAT albeit to a lesser extent than in WAT. Interest in the study of BAT physiology has been renewed by recent demonstration of considerable amounts of active tissue in many adult humans [47–50]. In adult knock-out mice, the size and appearance of BAT is similar to the wild-type animals [26]. At the molecular level, UCP1 expression, together with the expression of nuclear receptors PPARα, PPARγ, and fatty acid transporter aP2 is similar in both knock-out and wild-type animals. These findings suggested that BAT might not be a major site for RIP140 function or at least its lack of expression would not seem to have a big impact under basal conditions. Nevertheless, some recent experiments seem to point out a role for RIP140 in BAT. It has been shown that adult RIP140-null mice exhibit a reduced body core temperature and reduced mRNA expression of coregulator PGC1α in BAT, although response to an adrenergic activator does not seem impaired in these animals [51]. This is consistent with in vitro observations [21]. However, newborn and young RIP140-null mice exhibit a significant reduction in BAT mass and PGC1α mRNA expression, which might be associated with poor thermogenesis and that this in turn might account for the poor rate of postnatal survival [51]. More recently, in a cell line model of brown adipocytes, RIP140 was found to be recruited to and repress the CIDEA promoter through binding to both ERRα and nuclear respiratory factor (NRF) 1 [42]. Moreover, RIP140 has also been described to target and repress UCP1 enhancer in brown adipocytes trough LXR binding [23]. These findings suggest a role for RIP140 in BAT physiology, which might not be as evident as in WAT, but which could have important implication regarding energy expenditure. Further studies need to be carried out in both adults and newborn mice in order to establish the exact role of RIP140 in this tissue.

The amount of BAT and the expression of UCP1 correlate with increased basal metabolic rate and protection from obesity in both mice and humans. Genetic ablation of BAT induces obesity in mice [52]. Likewise, genetic deletion of UCP1 in mice causes weight gain when mice are kept at thermoneutrality [53]. On the other hand, ectopic expression of UCP1 in WAT results in resistance to obesity [54]. Mice with an increase in the amount of active BAT gain less weight and are more insulin-sensitive. Indeed, BAT mass and activity is greatly decreased in obese patients, particularly those with morbid obesity [50,55]. Therefore, the increased expression of genes in WAT from RIP140 null mice that are usually restricted to BAT seems to compromise the function of this tissue as the site of energy storage. Total energy consumption is increased significantly in RIP140-null mice, presumably as a consequence of energy dissipation in WAT resulting from the expression of UCP1 and increased mitochondrial activity [26]. BAT, on the other hand, utilises energy not only lipid oxidation but also glucose metabolism. Given the high metabolic capacity of BAT, any reduction in the amount or activity of BAT could perturb the ability of the body to dispose of glucose. Accordingly, the maintenance of a high glucose-utilizing activity by BAT could prevent the development of glucose intolerance and insulin resistance. As RIP140 has been found to control glucose metabolism in white adipocytes it will be of high interest to study its role in glucose metabolism in BAT. Glucose metabolism in BAT is under insulin and β-adrenergic control. β-Adrenergic responses were found to be unaltered, at least in primary cultures of brown adipocytes from RIP140 null mice [21]. It will be interesting to determine whether insulin signalling is affected when RIP140 levels are modified and whether high levels of RIP140 inhibit oxidative metabolism and expression of genes involved in thermogenesis, which could have a vast impact on the total energy expenditure in an individual.

## 3 Role of RIP140 in inflammation

Lipotoxicity and inflammation are intimately involved in the evolution of a number of metabolic diseases. Lipid overload of macrophages in vascular endothelium leads to phenotypic transformation into foam cells, secretion of pro-inflammatory cytokines, and apoptosis, contributing to atherosclerosis [56]. In steatotic liver, activation of kupffer cells, the resident macrophages, can drive hepatic inflammation and transition to steatohepatitis [57]. Accumulation of lipoproteins and lipid derivatives in the kidney induces macrophage infiltration and activation to initiate renal inflammation in chronic kidney disease [58]. Obesity in mice and humans is associated with a low-grade subclinical inflammation of adipose tissue [59–62]. Non-obese adipose tissue contains a quiescent population of local macrophages with little pro-inflammatory activity to support adipocyte function and maintain insulin sensitivity [63]. When hypertrophic, adipocytes secrete higher amounts of chemo-attractants to promote macrophage infiltration [9]. Additional macrophages may also be recruited simply to promote disposal of necrotic cells, which are more abundant in obese adipose tissue [30,64]. Free FA spill-over from enlarged adipocytes is able to activate Toll-like receptor (TLR) signalling and downstream Janus N-terminal kinase (JNK) and nuclear factor κB (NF-κB) pathways in the resident macrophages to elicit a switch towards a classically activated, pro-inflammatory phenotype [65,66]. The subsequent production of pro-inflammatory cytokines, including IL6 and TNFα, will interfere with insulin signalling leading to type 2 diabetes and further propagate the state of chronic inflammation [67,68]. The importance of inflammation in metabolic diseases has been reinforced by studies showing that limiting macrophage activation [67,69–71] and production of pro-inflammatory signals [72] is of value in reducing insulin resistance.

**Fig. 2:**
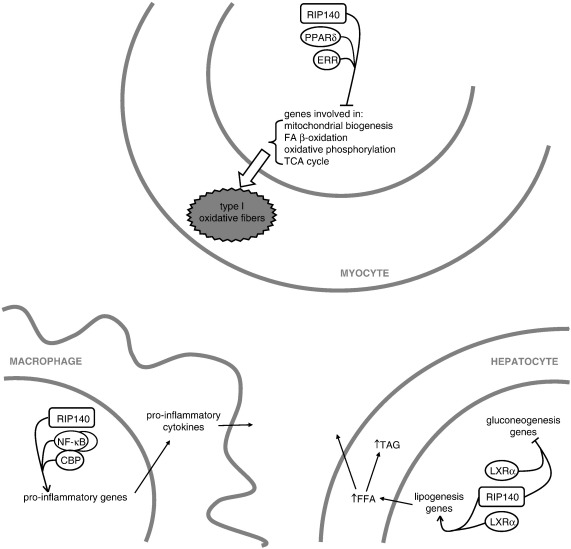
In muscle cells RIP140 functions as a corepressor for nuclear receptors to repress genes involved in oxidative metabolism. In macrophages RIP140 is a coactivator for NF-κB-dependent pro-inflammatory genes. In hepatocytes RIP140 is both a corepressor and coactivator for LXRα-dependent gene transcription to promote lipogenesis and reduce gluconeogenesis.

In contrast to the corepressor activities in adipose tissue and muscle, RIP140 can function as a coactivator in macrophages (Fig. 2). Comparison of gene expression profiles of macrophages derived from wild-type and RIP140 null mice has revealed that RIP140 deficiency impairs full execution of the pro-inflammatory program in response to TLR-mediated activation [29]. Downstream of TLRs, NF-kB is a major transcriptional regulator of pro-inflammatory gene expression, including TNFα, IL1β and IL6. RIP140 is recruited to these NF-κB-dependent promoters and stimulates transcription by acting as a bridging factor, stabilizing the formation of a trimeric complex with the RelA subunit and the CBP coactivator [29]. It is noteworthy that the absence of RIP140 causes only a partial attenuation of pro-inflammatory gene transcription and in cells where RelA expression is not limiting the coregulatory contribution of RIP140 might be modest [29]. Thus, the levels of transcription factors and other interacting cofactors can dictate the efficiency of a particular coregulator. This is an important concept when considering the inflammatory phenotype. RIP140 absence does not appear to immunocompromise mice [73,74] since pathogens provoke a strong immune reaction and in this context the function of RIP140 may have only a minor impact. However, in a more subtle state of inflammation, as found in obese adipose tissue, the absence of even a relatively weak activator might have a more pronounced outcome. Although insulin sensitivity in RIP140 null mice fed a high-fat diet is better than that in wild-type mice [37], the relative contribution of a blunted inflammatory response in macrophages relative to the altered lipid metabolism in adipose tissue, liver and muscle is unclear [22,26,75]. More insights would be provided by generation of mice with myeloid specific RIP140 deletion.

It is perhaps surprising that the expression of metabolic genes is essentially unaltered in macrophages in the absence of RIP140 in contrast to the situation in skeletal muscle and adipocytes [29]. These genes are regulated by PPARδ [75], PPARα and PPARγ [21] and LXRα [22], which play important roles in the control of lipid homeostasis and inflammation in response to endogenous fatty acid or sterol ligands. These NRs have been documented to mediate repressive effects on inflammatory genes in an NCoR- and SMRT-dependent manner [76–78]. It remains to be investigated if in macrophages, PPARs and LXRs can also form repression complexes with RIP140 on some genes, which would provide an interesting setting, where the coregulator has negative as well as positive functions.

Communications between adipocytes and macrophages are important in triggering inflammation in adipose tissue and it is increasingly recognized that both cell types contribute to the development of obesity-induced insulin resistance. Although the infiltrated macrophages are the main source of TNFα, adipocytes are responsible for a sizable fraction of the IL6 concentration in the circulation of obese patients [79,80]. The importance of an inflammatory response within adipocytes themselves is clear from a study with mice with adipocyte-specific JNK1 deficiency, which have improved insulin sensitivity in response to high-fat diet and normal levels of serum IL6 [67]. Interestingly, microarray interrogation of gene expression profiles shows that depletion of RIP140 in adipocytes causes genes encoding proteins involved in catabolic pathways for carbohydrates and fatty acids to be upregulated, but a subset of pro-inflammatory genes, including IL6, are downregulated (D. Morgenstein, unpublished observations;[39]). Therefore the activity of RIP140 as a coactivator of pro-inflammatory gene expression is unlikely to be restricted to macrophages and may also be found in adipocytes and possibly other cell types. This merits further investigation, for example, in vascular endothelial cells, where inflammation is the basis for atherosclerosis, or in the muscle, where macrophage infiltration and activation can give rise to muscle insulin resistance [81,82], and where copious amounts of IL6 are produced during exercise [83].

## 4 Role of RIP140 in muscle

### 4.1 Skeletal muscle

In both mouse and human, skeletal muscle contains distinct types of fibres, characterized by differential expression of specific myosin heavy chains. This difference in fibre composition accounts for different types of contractile function. Type I fibres (slow twitch fibres) are rich in mitochondria and show high levels of oxidative phosphorylation relaying mainly on fatty acid oxidation. These are the fibers resistant to fatigue and predominant in red muscles, such as the soleous. Type II (fast twitch fibers) contain a low number of mitochondria and relay more on anaerobic respiration via glycolysis or glycogenolysis that enable them to undergo rapid and short contractile bursts. In comparison to type I fibers, type II are more prone to fatigue. These types of fibers are predominant in muscles like the gastrocnemius and extensor digitorum longus (EDL) [84]. There is also an intermediate type of fibers, type IIX, that have fast twitch characteristics, but depend on oxidative metabolism that is similar to type I fibres [85]. Muscle fiber type composition can be modulated in response to several factors such as exercise, aging, hormonal changes or disease [86,87]. An increase in exercise has been shown to induce a fibre composition switch in the amount of type I fibres in muscles with predominantly Type II fibers such as the gastrocnemius. The switch in fiber type is reflected by changes in the expression of the myosin isoforms and other fiber type markers, as well as an increased expression of genes involved in mitochondrial myogenesis, fatty acid oxidation and oxidative phosphorylation. At a molecular level, a number of transcriptional regulators that are able to remodel skeletal muscle have been identified but it is still uncertain how the pathways are regulated. Nuclear receptors including PPARs, ERRα and other transcription factors such as CRE-binding protein (CREB), myocyte-specific enhancer binding factors (MEFs), activating transcription factors (ATFs) and NRFs have been found to activate expression of metabolic genes in skeletal muscle while the PGC1α plays a pivotal role as a coactivator for the aforementioned transcription factors [88,89].

Skeletal muscles play an important role in whole-body metabolism as they account for the 70% of the total insulin-stimulated glucose uptake. Alterations in its function or fibre composition can have a big impact on the whole-body metabolism. For example, in insulin-resistant states, such as obesity and type II diabetes, insulin-stimulated glucose uptake is markedly impaired [90], while increasing the number of type I fibres enhances insulin-mediated glucose uptake and protects against diabetes and other metabolic diseases [91]. Moreover, in obese patients, skeletal muscles have reduced metabolic oxidative capacities with morphologic changes that reveal decreased type I slow twitch fibers [92].

In skeletal muscle, RIP140 is expressed in a fiber type specific manner. RIP140 mRNA levels seem to be higher in glycolytic muscles, like gastrocnemius and EDL, rather than in oxidative muscles, like soleus and diaphragm [75]. In the absence of RIP140, EDL and gastrocnemius muscles exhibit a marked increase in mitochondrial activity accompanied by a corresponding shift in myofibers favouring the more oxidative type. Muscles appear redder in colour and express increased levels of type I fibres markers. These changes are accompanied by an increase in mitochondrial number and oxidative metabolism (most notably fatty acid oxidation) and are coordinated following upregulation of genes involved in these processes [75]. PPARδ and ERRα are direct targets for RIP140 action at least for some of the genes. Conversely, in transgenic animals, elevated RIP140 expression resulted in a decrease in both mitochondrial activity and the number of oxidative myofibers [75]. In contrast, muscles that consist of predominantly type I fibres (usually expressing low RIP140 levels) show an unaltered phenotype in the RIP140 null mice. This is consistent with the function of RIP140 as a corepressor and suggests a role for RIP140 as an inhibitor of oxidative metabolism in skeletal muscle [75] (Fig. 2). Considering the mass of skeletal muscle it can have an important impact on the whole-body energetic metabolism. It is conceivable that alterations in RIP140 expression may contribute to certain metabolic diseases such as obesity, insulin resistance and type II diabetes but this will require further investigation.

### 4.2 Cardiac muscle

Recent evidence suggests that impaired myocardial mitochondrial biogenesis, fatty acid metabolism, and antioxidant defence mechanisms lead to diminished cardiac substrate flexibility, decreased cardiac energetic efficiency, and diastolic dysfunction. These mitochondrial abnormalities can predispose to a metabolic cardiomyopathy and have been observed in insulin-resistant animal models and persons that are at higher risk of developing type 2 diabetes mellitus, hypertension, and cardiovascular disease [93].

RIP140 mRNA expression is high in cardiac muscle [26,94]. Studies in transgenic mice overexpressing RIP140 show that these mice are characterized by rapid onset of cardiac hypertrophy and ventricular fibrosis which results in an increase in the mortality rate from 4 weeks of age [26,94]. Interestingly, females are less sensitive to the deleterious effects of exogenous RIP140 expression compared to males, suggesting that estrogens might play a protective role against cardiac hypertrophy [95]. In these mice, overexpression of RIP140 leads to reduced expression of genes involved in fatty acid transport/oxidation and mitochondrial activity. This is accompanied by a decrease in mitochondrial number and activity, as demonstrated by decreased state III and state IV membrane potential and oxygen consumption, associated with abnormal morphology [94]. Thus, decreased energy production associated to increased fibrosis might be responsible for impaired cardiac function and decreased survival observed in the RIP140 transgenic mice. This study is consistent with data for skeletal muscle and myotubes obtained from RIP140-null mice where absence of RIP140 leads to increased oxidative metabolism [75]. Moreover, it highlights the importance of RIP140 in postnatal cardiac function and the possible implication in adult mitochondrial dysfunction leading to myopathies and cardiac malfunction.

## 5 Role of RIP140 in the liver

The hepatic manifestation of the Metabolic Syndrome is non-alcoholic fatty liver disease (NAFLD) and it is associated with insulin resistance although the cause and consequence relationship is debated [96,97]. NAFLD is diagnosed as the histological presence of lipid in more than 5% of hepatocytes, which is mostly in the form of TAG derived from the circulating pool of non-esterified fatty acids (59%), diet (15%) or de novo synthesis (26%) [98]. Hepatic lipogenesis is regulated by LXRα [99,100] and two studies have identified roles for RIP140 in this process. Our lab has found that in response to high-fat diet or agonist treatment, RIP140 is able to act as a coactivator for LXR-dependent induction of sterol regulatory element binding protein (SREBP) 1c and fatty acid synthase (FAS), which promote TAG synthesis [22]. Interestingly, RIP140 is also required for LXR-dependent repression of phosphoenolpyruvate carboxykinase (PEPCK) gene to limit gluconeogenesis in hepatocytes. Therefore, RIP140 and LXR can partner to form distinct activator or repressor complexes to either promote lipid generation or reduce glucose production, respectively [22] (Fig. 2). In contrast, a second study has shown that RIP140 contributed to hepatic steatosis during starvation and cancer cachexia by repressing SREBP1c [73]. In addition, RIP140 knockdown in the liver of starved mice altered hepatic FA oxidation, very low density lipoprotein (VLDL) production, FA uptake, serum TAG and peripheral LPL activity, to have a wider effect on local and systemic lipid homeostasis [73]. The reason for the contradicting results is not clear but differences in the experimental models used may have a contribution. We have utilized high-fat diet-induced liver steatosis with whole-animal RIP140 knockouts, as opposed to starvation or cachexia-triggered steatosis accompanied by shRNA-mediated liver-specific RIP140 knockdown. Nevertheless, both studies have found that RIP140 absence is beneficial by lowering hepatic TAG content in the given experimental context. Further experiments, perhaps making use of hepatocyte-specific RIP140-null animals, are required to understand the precise functions of the cofactor in this tissue.

## 6 Targeting RIP140 for treatment of metabolic syndrome

Lipotoxic events are the underlying cause of cellular damage in the metabolic syndrome and therefore tackling the oversupply of lipid to affected organs is important. Reducing caloric intake and increasing exercise to favour a negative energy balance should lead to clearance of ectopic lipid deposition and improve an array of metabolic parameters [101,102]. Nevertheless, although lifestyle changes are beneficial, obesity remains difficult to combat. A pharmacological approach is to increase the lipid storage capacity of subcutaneous adipose depots with a view to prevent overspill and accumulation in non-adipose organs. This is exploited by the thiazolidinedione (TDZ) class of drugs, which, by activating PPARγ, promote preadipocyte differentiation in addition to its insulin sensitizing effects [103]. Thus, the improved whole-body insulin sensitivity and reduced hepatic and visceral lipid content is attributed at least in part to the hyperplasic increase in subcutaneous adipose tissue seen in patients or animal models treated with TDZ [104–106]. In this case however, an improvement in metabolic well-being comes with increased obesity as a side-effect.

Excess fatty acids reaching non-adipose tissue can be activated to fatty acyl-CoA (FACoA) that undergo a series of esterification reaction to form lysophosphatidic acid (LPA), phosphatidic acid (PA), diacylglycerol (DAG) and finally TAG. Alternatively, FACoA can be channelled down the sphingosine synthesis pathway, where ceramide is an intermediate, or it can undergo β-oxidation to feed the mitochondrial electron transport chain [107]. All three fates have the potential to present the cell with toxic challenges. LPA, PA, DAG and ceramide can activate pro-inflammatory kinases to contribute to insulin resistance [108–111]. Indeed, ceramide levels are elevated in skeletal muscle of obese humans [112,113] and can further antagonize insulin signalling by inhibition of Akt [114]. Increasing the ability to oxidize FA, especially in the mitochondria of skeletal muscle, would reduce the accumulation of such intermediates and therefore protect from insulin resistance. It is important to note that several lines of evidence have questioned the role of mitochondrial function in insulin sensitivity and the cause and consequence relationship between the two remains highly debated [115]. Nevertheless, mitochondrial overload with lipid supply would increase reactive oxygen species (ROS) production, which has been shown to have a causal role in insulin resistance [116]. An important facet of treatment of metabolic disorders is limiting the damaging effects of the three possible fates of fatty acids, and RIP140 is involved in regulation of all three.

**Fig. 3:**
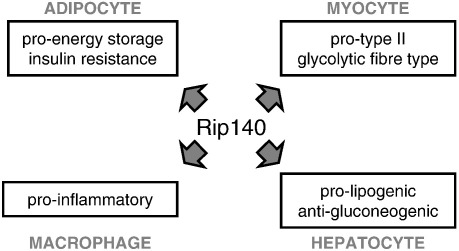
Actions of RIP140 in different metabolic tissue.

The pathways underlying the metabolic syndrome are interlinked in a complex network and an effective approach to treatment may need to target multiple points. For example, TDZ treatment, which activates PPARγ to reduce inflammation and promote adipogenesis and enhances adiponectin secretion to increase lipid oxidation [117,118], is often prescribed in conjunction with metformin to reduce gluconeogenesis in the liver, improve glucose uptake in peripheral tissue and overall insulin sensitivity. The evidence accumulated form studying RIP140 suggests that targeting the function of this cofactor for treatment of metabolic disorders would be of particular value since it would also have multiple beneficial consequences (Fig. 3). As detailed above, RIP140 null mice have a hypermetabolic phenotype, largely as a result of upregulation of genes involved in FA β-oxidation, oxidative phosphorylation, TCA cycle and mitochondrial biogenesis in adipose tissue and muscle [37,75]. This causes WAT to function in a manner akin to BAT to expend stored fat for thermogenesis, while in skeletal muscle the proportion of type I oxidative fibres, where TAGs are mainly used as fuel, is increased. Consequently, lipids are removed from the circulation, and both visceral and subcutaneous fat depots are reduced [26]. The upregulation of UCP1 in WAT [119] may also limit the amount of ROS produced by the mitochondria [120] to prevent inhibition of insulin signalling. Absence of RIP140 may have an additional protective effect on insulin signalling by preventing downregulation and inhibition of GLUT4 [43]. Impairing RIP140 function would protect against hepatic steatosis during a lipid challenge by promoting gluconeogenesis and reducing lipogenesis. Here, downregulation of SREBP1c and FAS restricts de novo FA synthesis [22], therefore reducing both TAG and sphingolipid biosynthetic pathways and the production of harmful intermediates. Furthermore, absence of RIP140 in macrophages would decrease the inflammation associated with obesity and insulin resistance [29]. The net results are improved lipid partitioning (decrease in circulating and ectopic load), decrease in adipose tissue size to allow it to regain normal lipid homeostatic function, and consequently, improved insulin sensitivity. The phenotype of RIP140 null mice is proof of principle that targeting RIP140 might constitute a successful approach for the treatment of metabolic syndrome. Although caution must be exercised since RIP140 function is essential for female fertility [28,121,122], this might not necessarily be a priority since metabolic disorders prevail at an age past sexual reproduction. Drugs seldom approach 100% efficiency, however, even a 50% inhibition of RIP140 activity would be beneficial, since heterozygous mice challenged with a high-fat diet also display improved metabolic parameters at a level intermediate between wild-type and null animals [74,75].

What pharmacological approaches could be used to modulate the function of RIP140? Three possibilities are outlined: (a) regulation of RIP140 expression, (b) regulation of RIP140 activity and (c) regulation of NR-dependent recruitment. In the first instance, several NR ligands have been shown to control RIP140 expression. In breast cancer cells estrogens [123], retinoic acid [124] and androgens [125] upregulate RIP140 mRNA levels thus providing potential feedback loops to limit nuclear receptor action. Estrogen antagonists may therefore be of value in preventing an induction in RIP140 expression [125], while progestins have been found to modestly downregulate RIP140 mRNA [126]. However, the use of such compounds would not offer efficient action in metabolic tissues that are largely non-responsive to steroid sex hormones. More interestingly, ERRs, which have been implicated in control of energy metabolism, can activate RIP140 gene expression by directly binding to an ERRE and indirectly through SP1 binding sites in the promoter [127]. ERRα-null mice have reduced body weight and peripheral fat deposits [128], reminiscent of the RIP140-null phenotype, yet the lack of a known ligand leaves modulation of its activity a difficult proposition. Recently miRNA have become very attractive tools in manipulating gene expression. The 5′ UTR of the RIP140 mRNA has been found to be targeted by microRNA (miRNA) mir-346. Rather than silencing RIP140 expression, mir-346 has been found to function in an atypical manner, increasing the protein levels and therefore the repressive activity of the cofactor, but a miRNA antagomir can be used to dampen RIP140 activity [129]. Furthermore, RIP140 mRNA contains a large 3′ UTR of ~ 3.5 kb that remains to be investigated for target sites of additional miRNAs.

In its role as a transcriptional regulator, RIP140 lacks any intrinsic enzymatic activities but is thought to function as a scaffold protein, providing a platform for the recruitment of enzymes such as C-terminal binding protein (CtBP) [130], histone deacetylases (HDACs) [131], DNA methyltransferese (Dnmt) [119], CBP [29], that change the accessibility of the promoter to the transcription machinery. Post-translational modifications (PTMs) lie at the basis of regulating the activity of non-enzymatic proteins. By favouring or disrupting specific interactions, PTMs can alter not only the recruitment of transcriptional complex partners but may also have wide-ranging consequences on the behaviour of proteins, including changes in subcellular localization and half-life. RIP140 is subject to a diverse array of PTMs. Multiple site of phosphorylation [132], acetylation [133], arginine [134] and lysine methylation [135] have been identified in RIP140 by mass spectrometry-based studies. Vitamin B6 [136] and SUMO conjugation [137] have also been described and may also modulate RIP140 function. Several PTMs may engage in an interplay to modulate RIP140 activity and affect cellular function. For example, PKCε-stimulated phosphorylation promotes PRTM1-directed arginine methylation to reduce RIP140 gene repressive activity by preventing interaction with HDAC3 and facilitating its export to the cytoplasm [134,138]. Conversely, MAPK-mediated RIP140 phosphorylation may enhance repressive activity [139,140]. Post-translational modifications are often investigated by ablating or mimicking them with the use of point mutations of modification sites. A caveat to such experiments is that a single amino acid can be subject to several modifications. For example, interpretation of results of a lysine mutant is complicated by the potential of the amino acid to undergo acetylation, methylation, SUMOylation or ubiquitination. However, if well validated, mechanistic knowledge of PTMs in RIP140 might be exploited for therapeutic purposes by targeting upstream signalling pathways that control its activity.

A third method of regulating RIP140 function involves taking advantage of its recruitment by NR. Partial agonists or antagonists may selectively prevent interaction with RIP140 while not interfering with other activities of the receptor. Since the metabolic repressor action of RIP140 is supported by NR-dependent recruitment, the discovery of such compounds would prove valuable for limiting the dampening effect on catabolism in adipose tissue and skeletal muscle. Notably, MRL24 has been recently identified as a PPARγ minimal agonist that inhibits Cdk5-mediated receptor phosphorylation to prevent obesity-associated gene dysregulation in fat cells [141]. Since chromatin occupancy of PPARγ was not altered, it is likely that differential recruitment of coregulators is responsible for the observed effect. Such a finding should encourage screening of ligands for PPARγ and other nuclear receptors for selective interaction with RIP140.

## 7 Conclusions

Overall this article summarizes the regulatory effects of RIP140 in tissues with an important role in the development and progression of the metabolic syndrome including white and brown adipose tissue, inflammatory cells, skeletal and cardiac muscles and liver. We have outlined signalling pathways that are subject to regulation by RIP140 and contribute to the development of the metabolic syndrome and speculated about the possibility of targeting RIP140 to ameliorate associated metabolic disorders.

In our opinion, therapeutic strategies targeting RIP140 have to be considered with caution, considering the broad spectrum of actions of RIP140 as well as its recently described role as a coactivator. Given that RIP140 is likely to be a difficult metabolic target to finely tune it would be necessary to screen for compounds that modify the recruitment of RIP140 to specific nuclear receptors at a subset of genes, or alternatively, compounds that can alter its binding to other components of the transcriptional machinery, such as histone modifying enzymes.
